# COVID-19 VACCINATIONS AMONG COMMUNITY-DWELLING PERSONS LIVING WITH DEMENTIA AND THEIR CAREGIVERS

**DOI:** 10.1093/geroni/igac059.2988

**Published:** 2022-12-20

**Authors:** Brandy Renee McCann, Jyoti Savla, Karen Roberto, Rosemary Blieszner

**Affiliations:** Virginia Tech, Blacksburg, Virginia, United States; Virginia Tech, Blacksburg, Virginia, United States; Virginia Polytechnic Institute & State University, Blacksburg, Virginia, United States; Virginia Tech, Blacksburg, Virginia, United States

## Abstract

There is a paucity of information regarding the uptake of the COVID-19 vaccine among community-dwelling persons living with dementia and their family caregivers. We examined factors influencing COVID-19 vaccine acceptance and hesitancy among community-dwelling persons living with dementia and their family caregivers during the early months of the vaccine rollout (February-March, 2021). Data came from three waves of telephone interviews (Mtime = 38.33 minutes) with 26 family caregivers living in rural Appalachian Virginia (96% White, 81% Female, Mage= 63±12 years, 42% Spouse Caregivers). We conducted a four-stage trajectory-based thematic content analysis and used the health belief model to interpret the data. Whereas all family caregivers and their relative living with dementia were eligible for the vaccine at Wave 3, only 10 dyads received it, while 10 dyads did not. In two families, the caregivers received the vaccine but not the person living with dementia; in four families, the caregiver had not received the vaccine, but their relative did. COVID vaccination acceptance was influenced by perceived direct and indirect health risks, cues from trusted allies, and ability to overcome vaccination barriers. Homebound people living with dementia faced unique barriers including transportation to vaccine sites and providers’ inability to deliver the vaccine to the home. Caregivers who refused or delayed vaccinations for themselves or their relatives discussed confusion about eligibility, low perceived risk of COVID, vaccine fear, and personal choice beliefs. Findings inform direct care delivery for interdependent family members who live in remote locations facing a public health crisis.

